# The value of oral contrast ultrasonography in the diagnosis of gastric cancer in elderly patients

**DOI:** 10.1186/s12957-018-1527-y

**Published:** 2018-12-07

**Authors:** Lan Liu, Dian-Yuan Lu, Jian-Rong Cai, Li Zhang

**Affiliations:** Department of Ultrasonography, Chongming branch of Xinhua Hospital Affiliated to Shanghai Jiao Tong University School of Medicine, No. 25 South Gate Road, Cheng Qiao Town, Chongming District, Shanghai, 202150 China

**Keywords:** Gastric filling ultrasound, Old age, Gastric cancer, Gastroscope

## Abstract

**Background:**

This study aims to investigate the value of oral contrast ultrasonography (OCUS) in the diagnosis of gastric cancer in elderly patients.

**Methods:**

OCUS data obtained from patients ≥ 60 years old were retrospectively analyzed and compared with gastroscopy results.

**Results:**

Among the 12,716 subjects examined by OCUS, 5021 subjects were ≥ 60 years old, which accounted for 39.48% (5021/12,716). Gastritis, gastric polyp, benign ulcer, and gastric cancer were detected by OCUS in 1099 patients. Among them, 196 patients underwent gastroscopy. Furthermore, ulcerative lesions were detected in 32 patients by OCUS and in 51 patients by gastroscopy, and the coincidence rate was 62.74%. Among these patients, gastric cancer was diagnosed in 18 patients by OCUS with a detection rate of 1.64% (18/1099) and detected in 19 patients by gastroscopy with a diagnostic coincidence rate of 94.73% (18/19). Furthermore, benign ulcer was detected in 14 patients by OCUS and in 32 patients by gastroscopy, and the diagnostic coincidence rate was 43.75% (14/32).

**Conclusion:**

OCUS helps to timely detect senile gastric cancer and can be used as a suitable technique for the detection of gastric diseases.

## Introduction

Gastric cancer is a prevalent malignancy in East Asia countries [1]. China has one of the highest incidence of gastric cancer in the world, and the overall survival rate is relatively poor [2]. The death rate of gastric cancer in the 2017 China annual report on cancer accounts for second. Hence, gastric cancer poses as a major threat to the health and life safety of people. Gastric cancer usually occurs in people > 50 years old. With the advent of the global aging society, the number of elderly patients with gastric cancer continues to increase. At present, the gold standard for the examination of gastric diseases is gastroscopy. However, due to age, physical condition, and other objective and psychological factors, the tolerance and compliance of elderly people to gastroscopy remain poor. Furthermore, there are many contraindications for gastroscopy. Hence, gastroscopy cannot be widely used as an appropriate method for the elderly. The body sensitivity of the elderly is decreased, and tolerance is enhanced. Hence, no specific symptoms and signs are exhibited. Therefore, gastric cancer is often diagnosed in the advanced stages. Aging has also become an important factor for the increase in the mortality of gastric cancer. In order to achieve good therapeutic effects and prognosis and improve the quality of life of patients [3, 4], early diagnosis and treatment of gastric cancer should be carried out for elderly patients. Therefore, a clinical examination suitable for the characteristics of elderly patients is urgently needed. In oral contrast ultrasonography (OCUS), the stomach is fully filled with liquids, in order to detect the lesions. The advantages of OCUS have been recognized, and the examination is becoming more mature. In the present study, we analyzed the value of OCUS in elderly patients. The specific analysis is as follows.

## Data and methods

### Subjects

A total of 12,716 symptomatic outpatients who received medical service in our department from January 2010 to December 2015 were included into the observation cohort. Among these patients, 5021 patients were ≥ 60 years old. Furthermore, among these patients, 1099 patients had positive ultrasound examination results, and the average age of these patients was 70.94 years old, with a maximum age of 93 years old. In the same period, among patients who were ≥ 60 years old, 196 patients underwent gastroscopy, and the average age of these patients was 69.51 years old, with a maximum age was 88 years old. This study was conducted in accordance with the Declaration of Helsinki. This study was conducted with approval from the Ethics Committee of Chongming branch of Xinhua Hospital Affiliated to Shanghai Jiao Tong University School of Medicine. Written informed consent was obtained from all participants.

### Instruments and methods

In the present study, GE Logiq E9, HD11, ALOKA color Doppler ultrasound diagnostic apparatus equipped with convex array probe was used, and the frequency was set at 2.5–3.5 MHz. The “Xinzhang” brand gastric window oral ultrasonic contrast agent (Zhejiang Hangzhou Huqing Yutang Pharmaceutical Co. Ltd.) was used.

#### Patient preparation

During the appointment, patients were instructed to avoid taking beans, milk, or other gas-producing foods; eat a light meal for dinner on the day before the examination; and water fast after dinner.

#### OCUS contrast agent preparation

Next, 48 g of gastric window oral ultrasonic contrast agent was evenly added into 300 ml of warm water, mixed into paste, placed for 10 min, and added and evenly mixed with 200 ml of hot water.

#### Examination methods

Generally, the patient was instructed to remain standing, with the physician facing the patient. The patient evenly takes 1/2 of the gastric filling agent, and the longitudinal section of the probe is placed under the xiphoid process of the patient, while slightly leaning to the left shoulder. After the cardiac orifice was revealed, patient evenly takes the residual 1/2 gastric filling agent, and the traffic condition of the cardiac orifice of the stomach is observed. Then, the probe is placed under the left costal arch and crosscut, continuous top-down scans are performed, the situations of the corpora ventriculi and gastric angle are successively observed, and the minimum position of the notch of the lesser curvature is determined. Next, the probe is clockwise adjusted into the longitudinal section and non-standard longitudinal section to observe the gastric antrum, pylorus, and duodenal bulb. After the duodenal bulb is filled 2–3 times, the examination is finished. Supplementary section: the fundus of the stomach is observed through the left intercostal section, and the gastric angle is observed through the chamfer section from the left paraumbilical region to the right shoulder.

### Diagnostic criteria

The diagnostic criteria are based on the “Practical Abdominal Diagnostics” (Second Edition) chief-edited by Haigen Cao and Jinrui Wang and the “Clinical application of Oral Gastrointestinal Contrast” chief-edited by Xinzhang Guo and Wu Zhang, which were mainly based on the sonographic diagnostic criteria of gastric diseases in gastroduodenal lesions.

## Results

**Fig. 1:**
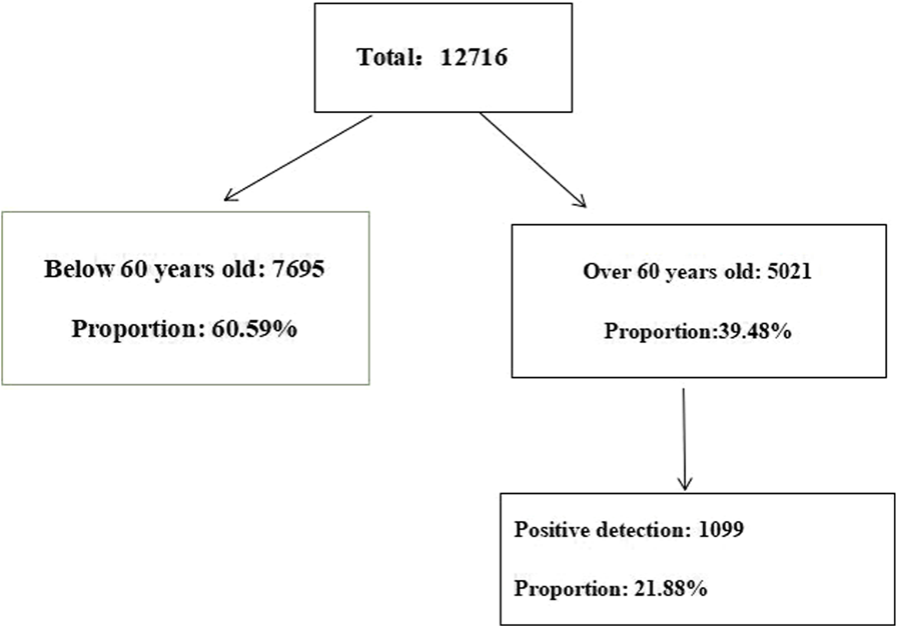
2010–2015 year gastric filling ultrasound examination for outpatients with symptomatic geriatric gastropathy

A total of 12,716 outpatients with symptomatic gastric disease, who underwent OCUS between 2010 and 2015, were included into this study. The average age of these patients was 70.94 years old, with a maximum age of 93 years old. The distribution of elderly subjects < 60 years old and ≥ 60 years old is shown in Fig. 1.

### Comparative analysis of results between OCUS and gastroscopy in elderly patients

A total of 196 elderly patients, who were ≥ 60 years old and had positive results by OCUS, underwent gastroscopy in the same period. Among these patients, 135 patients were male and 61 patients were female. The male-to-female ratio of these patients was 2.21:1.00, and the average age was 69.51 years old, with a maximum age of 88 years old. The comparative analysis of the diagnosis between OCUS and gastroscopy is shown. The detection rate of gastric cancer in OCUS was 9.18% (18/196), the detection rate of gastric carcinoma by gastroscope was 9.69% (19/196), and the accuracy rate of OCUS for diagnosis of gastric cancer was 94.73% (18/19). In detection of gastric cancer using OCUS, the sensitivity is 94.74%, the specificity is 100%, the positive predictive value is 100%, and the negative predictive value is 95%. The diagnostic results of OCUS were in good agreement with that of gastroscopy, Kappa = 0.972. The accuracy rate of various gastric diseases detected by OCUS is shown in Table 1.

**Table 1:** Comparative analysis of gastric filling ultrasonography and gastroscopy diagnosis ability

Pathological changes	Inspection method		Diagnostic accuracy of ultrasound (%)
	Gastric filling ultrasound	Gastroscope	
Gastric cancer	18	19	94.73
Benign ulcer	14	32	43.75
Gastric polyp	5	7	71.42
Stromal tumors	2	2	100
Benign gastric occupying lesion	1	1	100
Gastritis	56	135	41.48

## Discussion

Gastric cancer is the fourth most common cancer in the world and is the second leading cause of cancer-related deaths [5]. Gastric cancer has become a major public health burden. In recent years, domestic and foreign literatures have reported that the age of onset of gastric cancer has become older [6, 7], and its mortality rate has also increased with the increase in age [8]. The clinical diagnosis and treatment of elderly patients with gastric cancer has become a very urgent and realistic problem. Studies on the diagnosis and treatment of gastric cancer in the elderly have been reported at home and abroad [9, 10]. However, there are no studies on OCUS in the diagnosis of gastric cancer in elderly patients. Gastroscopy remains the main detection method and gold standard for gastric diseases. However, it brings about some pains and has contraindications. Due to age and physical factors, elderly patients cannot well tolerate the procedure and could not be easily examined. In the present study, among these symptomatic outpatient patients, patients who were ≥ 60 years old accounted for 39.48%, and the maximum age was 93 years old. From this, the requirement of suitable methods for gastric disease in elderly patients can be observed. OCUS was examined by filling the stomach with a food stomach contrast agent and forming a good ultrasonic interface with the stomach wall. OCUS can clearly display the five-layer structure of the gastric wall, lesions in gastric wall and their sizes, locations, and invaded layer of gastric wall. Clinicians can also observe the softness of the gastric wall through changes in the filling and emptying of the stomach. Its unique observation method is helpful for the further application of gastrointestinal ultrasound in clinic [11–14]. At present, the diagnostic value of OCUS in many common gastric diseases has been recognized, especially for peptic ulcer, gastric cancer, lymphoma, gastric polypoid lesions, and submucosal lesions [15–17]. When cutoff value of risk index based on OCUS parameters was set at 3 points, the sensitivity and specificity were 94.1% and 71.4%, respectively [18]. It is an effective supplement for gastroscopy and provides an option for elderly patients who cannot undergo gastroscopy. In this study, 196 cases were examined by OCUS and gastroscopy during the same period [18]. Cases of gastric cancer were diagnosed by OCUS, the detection rate was 9.18%, 19 cases were detected by gastroscopy, the detection rate was 9.69%, and the diagnostic accordance rate of ultrasound for gastric cancer was 94.73%. This is consistent with the results reported by Jun Lu and Chunmei Xu, in which the positive diagnostic rate of OCUS for gastric cancer was reported to be at 96.25% and 96%, respectively (Kappa = 0.972). This indicates that OCUS is an effective method for the detection of gastric cancer in elderly patients and can be used to substitute gastroscopy to detect gastric cancer in elderly patients, making up for the deficiency of gastroscopy in its application in elderly patients. Through observing changes in the thickness and the level structure and the function of local peristalsis of gastric wall, OCUS can provide many indices to indicate the presence of gastric cancer.

## Conclusions

The long-term survival rate of patients with progressive stages of gastric cancer is very low. Meanwhile, most patients with early gastric cancer can be cured or have a higher long-term survival rate after reasonable treatment. Furthermore, its 5-year survival rate is higher than 90%, and the prognosis is much better than that of patients with progressive stages of gastric cancer. In elderly people, due to the sluggish feel, the subjective symptom of gastric disease is mild or even absent. Furthermore, even if patients have symptoms, they are also not specific. Therefore, gastric cancer in elderly patients is often diagnosed in the late stages. In addition, elderly people have relatively weak body functions and more complications. Hence, operative mortality is higher than that of the other age groups. Therefore, elderly patients with gastric cancer should receive early diagnosis and treatment. OCUS has advantages of simple operation, no pain, and strong receptivity to the elderly. It has high accuracy in the diagnosis of gastric cancer. Our study shows that the ability of OCUS to detect and diagnose gastric cancer is similar to that of gastroscopy. It is proved that OCUS can be used as an effective means for early detection and diagnosis of gastric cancer in the elderly, and it can reduce the frequency of gastroscopy. OCUS is easy to learn and master, can be carried out in primary hospitals [19], and is worthy of promotion in primary hospitals, especially in community hospitals. After community doctors are trained with standardized operation, OCUS is expected to become a simple and effective screening and examination method for the prevention and treatment of gastric cancer in the community.

## Abbreviation

OCUS: Oral contrast ultrasonography
